# The influence of multidimensional deprivation on problem drinking developmental trajectory among young adults: a longitudinal study using latent class growth analysis

**DOI:** 10.1186/s13011-021-00426-2

**Published:** 2021-12-19

**Authors:** Soo Bi Lee, Sulki Chung

**Affiliations:** 1 Future Welfare Developing Human Resources for Community Innovation, Department of Social Welfare (BK21 FOUR), Jeonbuk National University, Jeonju, Republic of Korea; 2 Department of Social Welfare, Chung-Ang University, 84 Heuksuk-Ro, Dongjak-Gu, Seoul, Republic of Korea

**Keywords:** Deprivation, Social determinant of problem drinking, Housing, Problem drinking, Young adults

## Abstract

**Background:**

Many young people in Korea today experience deprivation in various areas of life. The social determinants of health approach maintains that social factors play an important role in an individual’s physical and mental health. This study aimed to investigate the problem drinking trajectory of young Korean people and identify the effects of multidimensional deprivation on problem drinking.

**Methods:**

The study used data from 2012 to 2018 found in the Korea Welfare Panel Study. Latent class growth analysis was performed to determine the number of trajectories of problem drinking. After identifying latent classes, a multinomial logistic regression analysis was utilized to examine multidimensional deprivation as a predictor of class membership.

**Results:**

Latent class analysis yielded three groups: (1) a low-level maintenance group (low level of alcohol use maintained at the low level), (2) a moderate-level increasing group (moderate level of problem drinking with a moderate increase in problem drinking), and (3) a risky drinking increasing group (high level of problem drinking with a rapid increase in problem drinking). Results from multinomial logistic regression showed that deprivation in housing and social deprivation increased the probability of belonging to the risky drinking increasing group compared to other reference groups.

**Conclusion:**

The study speaks to the need to establish appropriate intervention strategies according to the level and changes in the pattern of alcohol use. The implications of housing and social deprivation concerning problem drinking among young Korean people are also discussed.

**Supplementary Information:**

The online version contains supplementary material available at 10.1186/s13011-021-00426-2.

## Background

Problem drinking encompasses the level or potential of experiencing physical, emotional, and social problems due to alcohol use and overall social harm caused by drinking [1]. The social cost of problem drinking in Korea is estimated to be around 23 billion US dollars [2], and it is one of the major public health issues facing Korea today. Unhealthy drinking behavior has been associated with mental health problems such as depression and suicide and can lead to alcoholism, which involves decreased personal and social functioning and the breakdown of critical social ties [3–5]. According to the Organisation for Economic Co-operation and Development (OECD), the overall alcohol consumption in most countries has been declining in recent years, but risky drinking patterns such as high-risk and binge drinking have increased among young people and women [6]. A similar pattern has been observed in Korea, as reports have shown that the monthly binge-drinking rate among young people has increased to 46.6%, higher than that for other age groups. The rate of high-risk drinking among young people has also increased sharply to reach 15.6%, similar to that of the middle-aged group (15.7%), considered to be the group with the highest all-time prevalence of chronic problem drinking in Korean society [7]. Problem drinking among young people has been underestimated compared to that of other age groups in Korea, and recent trends point to the need for a closer examination of the matter.

Biological and psychosocial factors, developmental tasks, and the environments in which these tasks occur differ in each stage of the human life cycle. Several studies have indicated that drinking behaviors and associated harm are different according to each developmental stage [8, 9]. While targeting the entire adult population may still be necessary, to adequately understand problem drinking in the young population, we need to investigate the social circumstances they face. Adolescence is a period of professional and academic preparation for the future in which a young adult becomes independent from one’s family of origin and begins forming one’s own family [10]. Furthermore, it is a transitional period of becoming economically independent, making one’s own decisions, and being responsible for one’s deeds [11]. Therefore, young adults experience multiple events and face various crises while adapting to a new way of life, new social expectations, and new roles.

Inequality in Korea has been increasing since the economic crisis in the late 1990s that was followed by the aggravation of various social risks. Young Korean people today have a higher level of education than those of the past; however, they face higher unemployment, lower wages, and more precarious job situations regardless of individual efforts due to labor market instability. They also face unstable housing due to rising prices and economic pressures caused by student loans. As such, they are increasingly placed in unpredictable situations that hinder marriage, childbirth, and having a family [12, 13]. They experience physical and social deprivation in multiple aspects of life [14]. Young people are in a situation where it is difficult to meet the basic requirements for multiple dimensions of life. They are involuntarily experiencing deprivations in housing, work, health care, social activities, and social security.

This has resulted in young Koreans voluntarily classifying themselves into different socioeconomic classes based on “spoon discourse”^1^ (for instance, lower socioeconomic classes are referred to as “dirt spoons” versus those in the upper classes are referred to as being born with “gold spoons”). This reflects young people’s sense of shame and helplessness and symbolizes the deprivation felt by many of them [16–18]. Leading researchers who emphasize the social determinants of health have explained the various physical and mental health effects that occur when life opportunities are missing [19–21]. Social conditions such as the increase in inequality cause individuals to be more sensitive to both their social status and relative deprivation [19].

Relative deprivation experienced in daily life, a lack of social support, and a lack of control over one’s life play important roles in an individual’s physical and mental health including alcohol related problems [22]. According to the tension reduction hypothesis and alcohol expectancy theory, people are more likely to use alcohol or drugs to eliminate or control negative emotions when faced with distress [9, 23–25]. Factors such as low socioeconomic status, poverty, or deprivation of opportunities act as stressors [26], and there is an increased possibility of using alcohol or drugs to cope with depression or anxiety when such stressors are present. In particular, for young people who have limited resources, drinking can be an easily accessible coping method when they feel there is no other alternative that can help them cope with difficulties [27, 28].

In general, problem drinking is explained from a biopsychosocial perspective [29]. However, most studies examining alcohol-related problems among young Koreans have focused on psychological factors (i.e., depression and anxiety) [30, 31], and those that have explored environmental factors mainly centered on family and peer relationships [32, 33]. However, considering that young Koreans’ distress today is related to social factors, such as housing, job, and/or income problems, examining drinking-related problems in young people from the perspective of social determinants of health is essential [34–37]. Originating from research on health inequalities, the social determinants of health framework recognizes the power of socioeconomic factors as determinants of health [38]. It provides a foundation that considers the effects of relative disadvantages or deficits caused by social circumstances to explain problem drinking. One important social determinant affecting the lives of young people is relative deprivation. In this study, we apply Townsend’s concept of relative deprivation to examine problem drinking among young people. According to Townsend’s relative deprivation theory, poverty is defined as affecting those whose amount of resources are seriously below those of the average in a total population [39].

Recent studies have recognized the impact of deprivation and problem drinking on the Korean population [40–45]. However, most of these studies have targeted adults as a whole [37–39, 46, 47], the middle-aged [48], or the elderly [41, 45]. Likewise, studies of deprivation and problem drinking have largely examined social capital or deprivation only at the community level, and little attention has been paid to individual experiences of deprivation in multidimensional areas of life [20, 49–52]. Subsequently, there has been limited exploration of the effects of different types of deprivation as they relate to the problem drinking to which young people are sensitive. In addition, most studies of young people’s problem drinking have utilized cross-sectional analyses. Study results based on cross-sectional analyses are inapplicable to longitudinal predictions of the effects of deprivation on problem drinking [42–44].

Based on the discussion above, this study examines the influence of deprivation on the development of problem drinking trajectories in young people by utilizing a deprivation index that includes multidimensional deprivation. The purpose is to understand the longitudinal effects of deprivation in different areas on young people’s changing problem drinking patterns.

The specific research questions are as follows: (1) What are the trajectories for problem drinking among young people over time? (2) How does multidimensional deprivation among young people affect changes in the problem drinking patterns?

## Methods

### Data and sample

This study used data from the 2012 to 2018 Korea Welfare Panel Study compiled by the Korea Institute for Health and Social Affairs. Further details on the sampling design, methods, and data sets can be found elsewhere (www.koweps.re.kr) [53]. Consisting of data tracked annually since 2006, the Korea Welfare Panel Study is an ongoing longitudinal study of nationally representative Korean households containing individuals over 18 years old. The Study utilizes proportional systematic stratified cluster sampling to select participants [53]. For the present research, year 2012 was set as the base year, and seven time points were examined. The unit of analysis is the individual household member. A total of 1764 respondents aged between 20 and 39 from the 2012 Korea Welfare Panel Study were included in the analysis. The Korea Welfare Panel Study included weighted variables to correct for standard errors related to stratified cluster sampling and oversampling of lower socioeconomic classes. The weighted variables were included in all analyses to increase the generalizability of the study findings.

### Measures

#### Outcome variable: problem drinking

Problem drinking was measured using the Alcohol Use Disorder Identification Test (AUDIT). AUDIT is a screening tool developed by the World Health Organization (WHO) to screen problem drinking, and it consists of 10 questions. Each item is rated on a five-point scale, with the total score ranging from 0 to 40. Interpretations of the scale include using a cut-off score or using the total score as a continuous value. In this study, responses to 10 items were summed as a total score to classify potential groups of developmental trajectories for problem drinking. The higher the score, the higher the likelihood of problem drinking, which reflects dangerous and harmful drinking.

#### Predicting variable: multidimensional deprivation

Multidimensional deprivation was measured using the base year data from 2012. Seven areas of deprivation were measured. The experience of deprivation reflects the environment and socio-cultural climate in which one resides; therefore, we reviewed studies examining deprivation in Korea that utilized the same dataset [44, 47, 54–56]. We employed the index which was composed of the following seven sub-areas and 34 items: food deprivation (6 items), housing (10 items), education (2 items), work and income (4 items), social security (5 items), health and medical care (3 items), and social deprivation (4 items). The experience of each item was measured in a binary format (Yes =1, No = 0). Food deprivation included not having enough to eat and skipping meals due to financial difficulties. Deprivation related to housing refers to items such as housing costs, living environment, number of rooms, and residential space based on the official minimum residential standard [44, 54]. Educational deprivation included being unable to pay for minimum public education for more than a month and dropping out of school due to financial difficulties. Deprivation in work and income was defined as whether total cost of living exceeds the minimum cost of living, unemployment, working in physically harmful environments, and type of employment (i.e., precarious employment) [44, 55]. Deprivation in ​​social security included lack of national pension and health insurance, employment insurance, workers’ compensation, and severance pay and not making payments due to financial hardship [56]. Social deprivation consisted of having a family member with bad credit standing, inability to receive public services due to unpaid utility bills, and lack of family, social relationships, and support [44]. Finally, deprivation of health and medical care included not receiving medical services due to financial difficulties, not receiving appropriate care for chronic health problems, and dissatisfaction with health conditions [48].

#### Demographic variables

Demographic variables included gender (male = 0, female = 1), marital status (married, single, widowed/divorced/separated), religious status (yes = 0, no = 1), and residential area (metropolitan area = 1, non-metropolitan area = 0). Income level was categorized into low income (0) and other (1) based on 60% of the median income threshold, and education level was dummy coded as middle school or less, high school, and college or more.

### Statistical analysis

To identify the latent development trajectory classes (type of change) for problem drinking among young people and verify the influence of multidimensional deprivation on this pattern change, latent class growth analysis (LCGA) and multinomial logistic regression analysis were performed. LCGA, a form of growth mixture modeling (GMM), is useful for classifying latent classes over time within populations assumed to have similar characteristics [57]. Generally, the latent growth model assumes the pathways to be equal [58], even if there is heterogeneity within the group. However, latent class growth analysis can track heterogeneity within a group and can estimate different growth parameters for each latent class that shows different patterns of change. Therefore, it has the advantage of being able to identify latent classes according to the longitudinal change pattern of problem drinking, and at the same time, it can identify factors related to the classification of the class [59, 60]. Group-based methods estimate a finite number of subgroups, each having its own unique distinct behavioral sub-patterns. LCGA extends upon a conventional latent growth curve model (LGCM) to incorporate a categorical latent variable (i.e., classes) that represents a mixture of distinct subgroups. Unlike GMM, which assumes one identical growth curve by capturing an average trend, LCGA identifies different latent trajectory classes [59]. It helps to recognize latent subgroups with different initial intercepts or slopes and identify distinct patterns in the developmental trajectories in these groups [60].

Various criteria are used to determine the optimal number of latent classes in latent class growth modeling. The current study used Akaike’s information criterion (AIC) and the Bayesian information criterion (BIC) to determine the number of latent classes. In general, a smaller goodness-of-fit value indicates a better model, and the number of relevant latent classes and the proportion of subjects in each class are examined [59, 60]. These fit indices are sensitive to sample size; therefore, the final number of classes should be determined based on the interpretability of the classes according to the study objective and fit indices [61, 62]. The proportion of subjects belonging to *k* potential classes presented by the model should also be examined. Models with classes that include fewer than 5% of the samples can be excluded. After identifying latent classes with different problem drinking trajectories, multinomial logistic regression analysis was performed to identify areas of deprivation that predict these classes. Analyses were performed using STATA 16.0 [63].

## Results

### General characteristics

The general characteristics of the young adults in the base year 2012 data are presented in Table 1. Of the total respondents, 41.1% (*n* = 727) were male, and 59.0% (*n* = 1037) were female. A total of 1181 (72.3%) young people had a college degree or higher, and 26.9% (*n* = 560) had received a high school education. About half of the respondents (51.2%) were married, 47.3% (*n* = 721) were single, and 1.6% (38) were divorced/widowed/separated. Among the respondents, 51.9% (*n* = 731) lived in a metropolitan area, and 48.1% (*n* = 1033) lived in non-metropolitan areas. Using the 60% of the median income threshold, 6.5% (*n* = 155) of respondents were categorized as the low-income group.

**Table 1:** General characteristics of respondents

Variables	Categories	n or Mean
Gender	Male	727
	Female	1037
Education	Middle school	23
	High school	560
	College and higher	1181
Marital status	Married	1005
	Single	721
	Divorced/Widowed/Separated	38
Religion	Yes	772
	No	992
Residential Area	Metropolitan	731
	Non-Metropolitan	1033
Low Income (Poverty)	Non-poverty	1609
	Poverty	155
Deprivation	Total	3.758
	Food	0.04
	Housing	0.79
	Education	0.01
	Social security	0.54
	Work and income	0.68
	Social	0.97
	Health and medical care	0.24
Problem Drinking	Year 1 (2012)	4.21
	Year 2 (2013)	4.35
	Year 3 (2014)	4.27
	Year 4 (2015)	4.06
	Year 5 (2016)	4.29
	Year 6 (2017)	4.39
	Year 7 (2018)	4.55

### Correlation analysis

The zero-order relationship between independent and dependent variables was examined (see Additional file 1). The correlation coefficient ranged from 0.000 to 0.696, and the variance inflation factor (VIF) coefficient between variables was between 0.01 and 3.79, indicating that the data met the assumption of collinearity [64].

### Trajectory class of problem drinking

To determine the final optimal model, latent class growth analysis was performed. Table 2 presents the AIC and BIC of the models and the proportion of young people in each latent class. The growth model presented a total of five models. When fit indices and the proportions of the respondents in each class, along with the interpretability of the classes, were examined, the three-class model for the development trajectory of problem drinking was found to fit the data best. The proportions of young people within each class were 36.0% (*n* = 635) in class one, 45.6% (*n* = 804) in class two, and 18.4% (*n* = 325) in class three.

**Table 2:** Trajectory class of problem drinking

	Goodness of Fit		% by latent class				
	BIC	AIC	Class 1	Class 2	Class 3	Class 4	Class 5
1-class model	30,885.1	30,876.9	100.0				
2-class model	28,234.4	28,220.7	56.91	43.09			
3-class model	27,358.8	27,339.6	36.02	45.62	18.36		
4-class model	27,028.6	27,006.7	22.20	36.55	29.60	11.65	
5-class model	26,944.4	26,917.1	17.77	31.26	27.86	16.39	6.72

*AIC* Akaike information criterion

*BIC* Bayesian information criterion

**Fig. 1:**
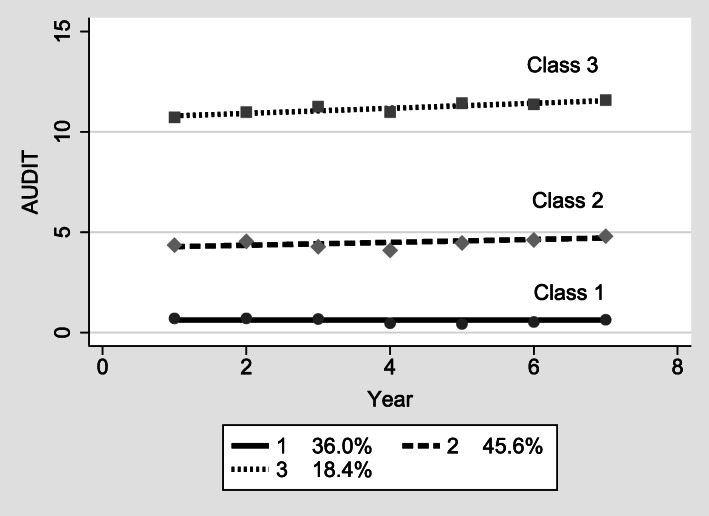
Development trajectories of latent classes of problem drinking. The figure depicts developmental trajectories of three latent classes of problem drinking identified.

The course trajectories and statistical significance of the three latent classes identified are presented in Table 3 and Fig. 1. The estimated values for the change pattern of each of the three classes were statistically significant. Three patterns in the developmental trajectories of problem drinking were extracted. The first latent class (Class 1), the low-level maintenance group, was composed of the group with the lowest level of problem drinking at the baseline (intercept = − 2.844, *p* < .001) and maintained this level over time (36% of respondents). In other words, no increase or decrease in problem drinking was detected longitudinally. The second latent class (Class 2), called the moderate-level increasing group, showed a moderate level of problem drinking initially (intercept = 3.773, *p* < .001) and exhibited a moderate increase over time (slope = .091, *p* < .01). Among the respondents, 45.6% were classified into this group. The third latent class (Class 3), named the risky drinking increasing group, showed the highest level of problem drinking at the beginning (intercept = 11.175, *p* < .001) and exhibited a rapid increase in problem drinking (slope = .125, *p* < .01). About 18% of the young people in the study fell into this category.

**Table 3:** Intercept and slope of each latent class

	%	Intercept		Slope		Name
		Estimate	S.E.	Estimate	S.E.	
Class 1	36.0	−2.844***	.574	–	–	Low-level maintenance group
Class 2	45.6	3.773***	.554	.091**	.038	Moderate-level increasing group
Class 3	18.4	11.175***	.629	.125**	.041	Risky drinking increasing group

*p < .05, **p < .01, ***p < .001

### Effects of deprivation on identified course trajectories of problem drinking

To analyze the effects of the predictive factors of the three latent class memberships, multinomial logistic regression analysis was performed using two reference groups: the low-level maintenance group (Class 1) and the moderate-level increasing group (Class 2). The model indicated a good fit (LR Chi^2^ (30) = 278.78, *p* = .000), and the explanatory power of the model (Pseudo R^2^) was .155 (Table 4).

**Table 4:** Effects of deprivation on problem drinking: Multinomial logistic regression analysis

	Low-level maintenance group				Moderate-level increasing group	
	Moderate-level increasing group		Risky drinking increasing group		Risky drinking increasing group	
	RRR	95% CI	RRR	95% CI	RRR	95% CI
Constant	3.708	0.986–13.967	4.889	0.612–39.06	1.319	0.186–9.352
Gender (Male)						
Female	.383^***^	0.275–0.531	.034^***^	0.021–0.055	.088^***^	0.056–0.139
Education (Middle school)						
High school	.840	0.232–3.040	.919	0.118–7.178	1.094	0.157–7.607
College and higher	.687	0.193–2.451	.353	0.046–2.723	.514	0.075–3.547
Marital status (Married)						
Single	1.755^***^	1.303–2.362	1.272	0.822–1.967	.725	0.492–1.069
Divorce/widow/separated	.570	0.789–1.725	1.007	0.280–3.622	1.766	0.610–5.113
Religion (No religion)						
Have religion	.573^***^	0.433–0.759	.437^***^	0.290–0.658	.761	0.521–1.112
Low income (Low income						
Not low income	1.125	0.651–1.943	.355^*^	0.157–0.803	.316^**^	0.145–0.688
Residence (Non-metropolitan)						
Metropolitan	.998	0.755–1.320	1.049	0.703–1.565	1.052	0.728–1.519
Deprivation in						
Food	.793	0.479–1.312	.769	0.333–1.773	.970	0.451–2.084
Housing	.973	0.860–1.102	1.077^*^	1.005–1.155	1.107^*^	1.034–1.254
Education	.945	0.281–3.183	.299	0.027–3.341	.317	0.040–2.492
Social security	1.024	0.901–1.164	.933	0.758–1.149	.911	0.749–1.108
Work and income	.730^*^	0.552–0.966	.595	0.407–0.872	.815	0.574–1.159
Social	1.201	0.908–1.588	1.712^**^	1.181–2.481	1.425^*^	1.007–2.017
Health and medical care	1.029	0.739–1.432	1.382	0.913–2.091	1.343	0.923–1.954

*N* = 1764, LR Chi^2^(30) = 278.78, *p* = .000, Pseudo R^2^ = .155; *p < .05, **p < .01, ***p < .001; RRR = Relative Risk Ratio; CI = confidence interval

First, the relative risk ratios (RRR) of the moderate-level increasing group (class 2) and risky drinking increasing group (class 3) were examined using the low-level maintenance group (class 1) as the reference group. Compared to the low-level maintenance group, being female (RRR = .383, RRR = .034, *p* < .001) and having religion (RRR = .573, RRR = .437, p < .001) were less likely to belong to both classes 2 and 3. Compared to the low-level drinking group, single young people were more at risk of belonging to the moderate-level drinking group with an increase in problem drinking over time (RRR = 1.755, p < .001). Compared to the low-level drinking group, a low income or poverty predicted young people falling into the risky drinking group with an increase in risky drinking longitudinally (RRR = .355, *p* < .05).

In the context of multidimensional deprivation, none of the deprivation areas experienced by young people predicted moderate-level problem drinking compared to the low-level problem drinking group. However, young people who experienced housing deprivation and social deprivation were more at risk of belonging to the risky drinking increasing group compared to the reference group (Class 2). The findings indicate that compared to young people in the low-level maintenance group, housing deprivation increases the probability of engaging in risky drinking by 1.077 times (RRR = 1.077, *p* < .01), and those who experience social deprivation are 1.712 times more likely (RRR = 1.712 *P* < .01) to engage in risky drinking that will increase over time.

Next, predictors of Class 3 (risky drinking increasing group) were analyzed using Class 2 as the reference group. The results indicated that women (RRR = .088, *p* < .001) and those not in poverty (RRR = .316, *p* < .01) were less likely to belong to Class 3. In other words, men, and those in the low-income group are more at risk of engaging in problem drinking that continues to grow over time.

Analysis of multidimensional deprivation factors showed that housing and social deprivation are significant predictors of belonging to the Class 3. Compared to the moderate problem drinking group, those who experience deprivation in housing and social areas were 1.107 times (RRR = 1.107, *p* < .05) and 1.425 times (RRR = 1.425, *p* < .05) more at risk for engaging in risky drinking longitudinally. The findings indicate that housing and social deprivation among young people are significant predictors of problem drinking.

## Discussion

Today, many young people in Korea experience deprivation in various areas of life while carrying out developmental tasks in the human life cycle. Previous research indicated that such experiences act as social determinants that affect problem drinking. The present study used LCGA and multinomial logistic regression analysis to investigate the effects of multidimensional deprivation experienced by young people on the developmental trajectories of problem drinking. This was examined using nationally representative longitudinal data. By employing LCGA, we identified multiple longitudinal patterns in problem drinking. The classification of distinct subgroups, each having a similar pattern of trajectories, allowed us to identify class membership and examine predictors of problem drinking varying across classes.

The latent group analysis of problem drinking’s developmental trajectory among young people, i.e., the type of change over time, yielded three latent groups. The first group called the low-level maintenance group (36.0%), comprises Korean young adults who had low levels of alcohol use and maintained this low level during the study period. The second group, called the moderate-level increasing group (45.6%), includes young adults who initially showed moderate levels of problem drinking and exhibited a moderate increase in problem drinking over time. The last group, called the risky drinking increasing group (18.4%), consists of young people who had the highest level of problem drinking at the baseline and showed a rapid increase in problem drinking. These developmental trajectories among young people show that there are different patterns of problem drinking development. Previous studies focused on alcohol problems among young people in general [65, 66]; however, the current study expands on previous knowledge by identifying different longitudinal patterns in young people’s drinking in Korea.

These findings suggest that prevention and intervention programs should consider different levels of problem drinking among young people. For instance, for young people who fall within low-level problem drinking, prevention efforts should focus on keeping alcohol use at this low level so that they do not develop a risky drinking habit. Drinking patterns formed during early adulthood are likely to persist in later life [23, 67, 68]. This is especially true in Korea, as it is known as a culture of high tolerance toward drinking behavior. Prevention education, early counseling programs, and local campaigns geared toward young people and college students should be implemented to foster healthy drinking habits. Early screening and interventions, including moderate drinking programs, should be provided for those in the risky drinking group. For those in need of treatment, a system that provides appropriate referrals is necessary. Harmful drinking at a young age may indicate the beginning of chronic alcohol problems; therefore, active interventions are required for this group.

The findings from multinomial logistic regression indicated that being male, poverty, housing deprivation, and social deprivation were significant predictors of belonging to the risky drinking increasing group. Although the effects were statistically significant, it is necessary to consider the effect size of these factors. Examination of the relative risk ratios showed that being male and being in poverty had a larger influence on belonging to the risky drinking increasing group than social deprivation and housing deprivation. This finding is consistent with previous knowledge, which had consistently reported that men experience more drinking problems than women. The fact that poverty, a main socioeconomic indicator, has the most important role in predicting risky drinking confirms the importance of examining social determinants of alcohol related issues. Poverty is closely related to many areas of deprivation. If practice and policy on alcohol-related problems do not take livelihoods into account, the benefits of intervention effects will be limited.

Examining the effects of multidimensional deprivation on the latent classes of developmental trajectories of problem drinking among young Korean people, the study results indicate that deprivation in housing and social deprivation increased the probability of belonging to the risky drinking increasing group compared to each reference group (the low-level maintenance group and the moderate-level increasing group). The association between housing deprivation and the change in pattern in problem drinking is consistent with previous studies that maintained that housing is a significant factor of alcohol-related behavior [37, 69]. This also supports studies that have reported that the residential environment is a predictive factor of physical and mental health including problem drinking [70, 71]. For example, one longitudinal study found that the experience of housing deprivation increased the risk of problem drinking [37]. Current findings point to the need to examine the meaning of housing in Korean society. Correlation analysis shows that living in a metropolitan area and housing deprivation are negatively associated (Additional file 1). This finding seems counterintuitive, considering the high price of housing in metropolitan areas. However, housing deprivation does not only include housing prices, but also includes the living conditions of the residential area, ability to pay rent, structure of the place, and so on. In other words, the negative relationship between residential area and housing deprivation may mean that young people living outside the metropolitan area may be in a worse economic position and may have relatively low satisfaction with their living environment (i.e., low accessibility to various resources and activities). When both variables are included in the analyses, only housing deprivation was a significant predictor of problem drinking.

A residence is a basic living condition in life, but housing has various meanings for young Koreans. Having the means to secure appropriate housing is considered an essential condition for completing developmental tasks, such as marriage and having children, and a way to relieve anxiety about uncertainties in life [12, 13, 54]. Housing has become an emblematic example of inherited wealth in the country. As a result, housing has become a distinctive indicator of inequality and a proxy for success [54]. Unlike older generations who were able to achieve homeownership through individual efforts and hard work, young people who face current social conditions, such as the decrease in decent job opportunities, income polarization, and increasing housing prices, perceive housing to be a large factor exacerbating their sense of deprivation.

Ultimately, housing deprivation reflects inequality for young people, and we may infer that this perceived inequality has contributed to a sharp increase in problem drinking [35]. The provision of stable housing is known to assist in the recovery from alcoholism [72, 73]. The current study’s findings, along with the results from several previous studies, confirm that the physical environment, such as stable housing, has a direct and indirect effect on mental health, and specifically, problem drinking. This implies that we need to consider the physical environment in initial assessments and during the creation of interventions for problem drinking. Interventions may have to include referrals to resources for housing services when possible. Outreach and pop-up counseling booths for early screening and brief interventions may benefit so-called “one-room villages” where many vulnerable young people in Korea reside. The physical environment, such as housing, is not usually considered a factor associated with problem drinking, but our study showed that housing issues affect it.

Finally, the study findings reported on the relationship between social deprivation and problem drinking among young people, indicating the social environment young people face today. With the growing instability in the labor market, young Koreans are repeatedly entering and exiting different jobs to seek better placements [74]. Young people in this situation may have great desires for self-development, social relationships, and cultural life [75]; however, they might still experience limited opportunities to socialize with others and are involuntarily excluded from resources that make emotional and social connections possible [14]. The social relationships that act as a protective factor for problem drinking may be weakened. The common factor underlying both housing and social deprivation is anxiety. Social deprivation is related to feeling alienated and disconnected from others, which leads to anxiety. When people are disconnected from the opportunities that compose the standard of a happy life in that society, they are likely to experience low self-esteem and shame due to deprivation and exclusion, which may eventually be expressed in the form of social pathologies, including problematic alcohol use [76]. In other words, addictions are used to adapt to feeling alienated and disconnected. In particular, for young people with limited resources, alcohol can act as a quick remedy or coping method for anxiety.

In addition, there is an argument that young people lack formal social welfare resources compared to other generations [74]. There are relatively fewer public resources for young adults who may require services when facing economic or psychological crises and social deprivation. Therefore, we can infer that those vulnerable to social deprivation are more likely to experience risky drinking and continue to engage in risky drinking over time. Early interventions are needed to detect and prevent alcohol-related problems for those likely to follow high-risk trajectories.

This study’s findings suggest that public policies targeting young people should give more attention to their problem drinking and seek ways to expand and improve public social networks and social resources. Comparing the results of this study with change patterns of problem drinking of those in the middle-aged and older generations, we can observe several patterns of problem drinking as well as areas of social deprivation that affect it. A recent study that used similar data showed that for the middle-aged group, social security, work, and income deprivation were significant predictors of problem drinking, whereas social deprivation was the main predictor for the elderly group [77]. For young people, policies and programs should be designed to provide various social coping resources that help prevent disconnections from family and friends and expand social and emotional exchange. Young people living alone with unstable jobs are particularly at risk of social deprivation and may benefit from monitoring services that can detect problem drinking and other mental health issues.

This study examined a longitudinal relationship between deprivation and problem drinking using a deprivation index based on different needs in the stages of the human life cycle. Through the study, we were able to identify the social risks young people face today in the process of performing developmental tasks and how these risks affect individual mental health, specifically problem drinking. This study is of value in that it examined specific areas of deprivation to which young people are more sensitive and their association with drinking practices. Furthermore, this work adds to current knowledge in alcohol research by examining longitudinal changes in groups showing different patterns of developmental trajectories.

Despite the contributions of this study to the literature on alcohol problems, it is important to identify its limitations. The deprivation index was necessarily formed only from items available in the limited secondary data, and it could not include all the functional and diverse areas of deprivation young people may face in daily life today. Another limitation of the data is that they are correlational, and it is difficult to determine a causal relationship between independent variables. Finally, the study only examined the trajectories of problem drinking and did not follow changes in multidimensional deprivation over time. Because longitudinal changes in deprivation as predictive factors can influence problem drinking, future research should examine changing patterns in different areas of deprivation to understand the more dynamic processes in the relationship.

## Conclusions

This study examined changes in problem drinking among young Korean people over seven years using the Korea Welfare Panel Study to classify latent classes with different problem drinking trajectories. Furthermore, this study examined how various dimensions of deprivation predict latent class membership. Three latent classes with different trajectories that reflect changing patterns in problem drinking were identified: the low-level maintenance group, the moderate level increasing group, and the risky drinking increasing group. This study provides evidence for the need to establish appropriate intervention strategies according to the level of and patterns of change in alcohol use. The study results also confirmed that deprivation, a relative disadvantage or deficiency, experienced in society influences young people’s level of problem drinking and the patterns of change over time. Specifically, Korean youngsters who are deprived of housing and social connections are more likely to engage in higher levels of problem drinking and exhibit increased risky drinking over time. Drinking practices and high-risk drinking may be an individual’s choice. Still, our study confirmed the role of social factors such as housing and social deprivation, known as the social determinants of health, on problem drinking.

## Supplementary Information

**Additional file 1. **Correlation among the study variables. The table describes correlation coefficients between major variables. * *p* < 0.05; 1 problem drinking; 2 gender (male); 3 age; 4 education; 5 marital status (single); 6 marital status (divorced/widowed/separated); 7 residential area (metropolitan); 8 low income; 9 religion; 10 food deprivation; 11 housing deprivation; 12 education deprivation; 13 work and income deprivation; 14 social security deprivation; 15 social deprivation; 16 health and medical care deprivation.

## Data Availability

The datasets used and analyzed during the current study are available from the corresponding author on reasonable request.

## Abbreviations

AIC: Akaike’s information criterion
AUDIT: Alcohol use disorders identification test
BIC: Bayesian information criterion
GMM: Growth mixture modeling
LCGA: Latent class growth analysis
LGCM: Latent growth curve model
RRR: Relative risk ratio
VIF: Variance inflation factor
WHO: World Health Organization

## Footnotes

1 “Spoon discourse” is presumed to have been derived from the expression “born with a silver spoon in one’s mouth” [15].
