# Supplementary material for: The influence of multidimensional deprivation on problem drinking developmental trajectory among young adults: a longitudinal study using latent class growth analysis
Source: Subst Abuse Treat Prev Policy. 2021 Dec 19;16:90. doi: 10.1186/s13011-021-00426-2 (PMC8684624; doi:10.1186/s13011-021-00426-2)
Supplement: Supplementary file 1 — Additional file 1. Correlation among the study variables. The table describes correlation coefficients between major variables. * p < 0.05; 1 problem drinking; 2 gender (male); 3 age; 4 education; 5 marital status (single); 6 marital status (divorced/widowed/separated); 7 residential area (metropolitan); 8 low income; 9 religion; 10 food deprivation; 11 housing deprivation; 12 education deprivation; 13 work and income deprivation; 14 social security deprivation; 15 social deprivation; 16 health and medical care deprivation. [file 13011_2021_426_MOESM1_ESM.docx]

**Additional File 1. Correlation among the study variables**

|  | 1 | 2 | 3 | 4 | 5 | 6 | 7 | 8 | 9 | 10 | 11 | 12 | 13 | 14 | 15 |
| --- | --- | --- | --- | --- | --- | --- | --- | --- | --- | --- | --- | --- | --- | --- | --- |
| 1 | 1 |  |  |  |  |  |  |  |  |  |  |  |  |  |  |
| 2 | .512* | 1 |  |  |  |  |  |  |  |  |  |  |  |  |  |
| 3 | .031 | .119* | 1 |  |  |  |  |  |  |  |  |  |  |  |  |
| 4 | -.107* | .057* | -.170* | 1 |  |  |  |  |  |  |  |  |  |  |  |
| 5 | .050* | .091* | -.696* | .184* | 1 |  |  |  |  |  |  |  |  |  |  |
| 6 | .059* | .051* | .098* | -.070* | -.121* | 1 |  |  |  |  |  |  |  |  |  |
| 7 | -.166* | -.075* | -.019 | .091* | .045 | -.055* | 1 |  |  |  |  |  |  |  |  |
| 8 | -.037 | .041 | -.061* | -.080* | .083* | .091* | -.045 | 1 |  |  |  |  |  |  |  |
| 9 | .046 | .023 | .031 | .034 | -.001 | -.033 | -.023 | -.068* | 1 |  |  |  |  |  |  |
| 10 | -.032 | -.016 | -.049* | -.029 | .033 | .005 | .004 | .224* | -.008 | 1 |  |  |  |  |  |
| 11 | .055* | .035 | -.041 | -.147* | .034 | .054* | -.059* | .218* | .091* | .132* | 1 |  |  |  |  |
| 12 | -.033 | -.036 | -.113* | .010 | .105* | -.015 | .028 | -.031 | .055* | .169* | .061* | 1 |  |  |  |
| 13 | .038 | .046 | -.049* | -.087* | .092* | .091* | .001 | .026 | .023 | .018 | .073* | .108* | 1 |  |  |
| 14 | -.132* | -.146* | -.152* | -.155* | .075* | .029 | -.033 | .187* | -.061* | .110* | .101* | .005 | .084* | 1 |  |
| 15 | .043 | -.048* | -.007 | -.026 | .019 | .066* | .049* | .073* | -.006 | .113* | .103* | .023 | .082* | -.002 | 1 |
| 16 | .047* | .033 | .047* | -.100* | .000 | .061* | -.076* | .147* | .007 | .131* | .139* | -.015 | .004* | .104* | .027 |

* p < 0.05; 1 problem drinking; 2 gender (male); 3 age; 4 education; 5 marital status (single); 6 marital status (divorced/widowed/separated); 7 residential area (metropolitan); 8 low income; 9 religion; 10 food deprivation; 11 housing deprivation; 12 education deprivation; 13 work and income deprivation; 14 social security deprivation; 15 social deprivation; 16 health and medical care deprivation
